# Pregnancy recognition signaling mechanisms in ruminants and pigs

**DOI:** 10.1186/2049-1891-4-23

**Published:** 2013-06-26

**Authors:** Fuller W Bazer

**Affiliations:** 1 Department of Animal Science, Texas A&M University, College Station, 442D Kleberg Center, 2471 TAMU, Texas 77843-2471, USA

**Keywords:** Pregnancy, Interferon Tau, Estrogen, Prostaglandin F_2α_

## Abstract

Maternal recognition of pregnancy refers to the requirement for the conceptus (embryo and its associated extra-embryonic membranes) to produce a hormone that acts on the uterus and/or corpus luteum (CL) to ensure maintenance of a functional CL for production of progesterone; the hormone required for pregnancy in most mammals. The pregnancy recognition signal in primates is chorionic gonadotrophin which acts directly on the CL via luteinizing hormone receptors to ensure maintenance of functional CL during pregnancy. In ruminants, interferon tau (IFNT) is the pregnancy recognition signal. IFNT is secreted during the peri-implantation period of pregnancy and acts on uterine epithelia to silence expression of estrogen receptor alpha and oxytocin receptor which abrogates the oxytocin-dependent release of luteolytic pulses of prostaglandin F2-alpha (PGF) by uterine epithelia; therefore, the CL continues to produce progesterone required for pregnancy. Pig conceptuses secrete interferon delta and interferon gamma during the peri-implantation period of pregnancy, but there is no evidence that they are involved in pregnancy recognition signaling. Rather, pig conceptuses secrete abundant amounts of estrogens between Days 11 to 15 of pregnancy required for maternal recognition of pregnancy. Estrogen, likely in concert with prolactin, prevents secretion of PGF into the uterine venous drainage (endocrine secretion), but maintains secretion of PGF into the uterine lumen (exocrine secretion) where it is metabolized to a form that is not luteolytic. Since PGF is sequestered within the uterine lumen and unavailable to induce luteolysis, functional CL are maintained for production of progesterone. In addition to effects of chorionic gonadotrophin, IFNT and estrogens to signal pregnancy recognition, these hormones act on uterine epithelia to enhance expression of genes critical for growth and development of the conceptus.

## Introduction

### Type I and type II interferons

Interferons are cytokines with antiviral, antiproliferative and immunomodulatory biological effects critical to immune responses that protect the body against viral infections and malignant cells [1]. Type I interferons with a high degree of structural homology include interferons alpha (IFNA1-IFNA10, IFNA13, IFNA14, IFNA16, IFNA17 and IFNA21), interferon beta (IFNB), interferon delta (IFND), interferon epsilon (IFNE), interferon kappa (IFNK), interferon tau (IFNT) and interferon omega (IFNW1-IFNW3). IFNT is unique in being the pregnancy recognition signal in ruminants. The IFNT family of proteins are structurally and functionally related to each other and to other type I interferons and IFNT likely arose from duplication of an IFNW gene some 36 million years ago when IFNT came to be expressed in the trophectoderm under control of an Ets-2/AP-1 enhancer element [2]. IFNT is expressed only by mononuclear trophectoderm cells of ruminant conceptuses (embryo and its extra-embryonic membranes). IFND, another novel type I interferon is expressed by conceptuses of pigs and horses during the peri-implantation period of pregnancy [3]. Interferon gamma (IFNG) is a type II interferon secreted by pig conceptuses during the peri-implantation period of pregnancy [3]. The functions of IFND and IFNG from pig conceptuses are not known [4-6].

Type I IFNs bind a common receptor composed of IFNAR1 and IFNAR2 to induce cell signaling via the Janus activated kinases (JAKs) and tyrosine kinase 2 (TYK2) pathway [1,7,8]. Type I IFNs also induce formation of signal transducer and activator or transcription homodimers (STAT1–STAT1) known as gamma-activation factor (GAF) that translocate to the nucleus and bind GAS (gamma-activation site) elements in the promoter region of interferon stimulated genes (ISG). One GAS-regulated gene is interferon regulatory factor *1(IRF1)* which binds and activates interferon stimulated response elements (ISREs) of many ISG to amplify effects of type I IFNs [9,10]. However, type I IFNs act predominantly via interferon stimulatory gene factor 3 gamma (ISGF3G) rather than GAF. The ISGF3G complex includes the STAT1: STAT2 heterodimer and IRF9. The predominant cell signaling pathways involve *STAT2* and *ISGF3G* that prolong effects of IFNT by increasing expression of STAT2 and IRF9 which favors formation of ISGF3G rather than GAF [11,12]. However, type I IFNs also activate non-classical cell signaling pathways that include mitogen activated protein kinases (MAPKs), especially p38 and ERK1/2, as well as the phosphatidyl inositol kinase 3 kinase (PI3K)/V-AKT murine thymoma viral oncogene homolog 1 (AKT1) pathway and mechanistic target of rapamycin (MTOR) [1,13].

IFNG is critical for innate and adaptive immunity against viral and intracellular bacterial infections and tumor control, as well as activating macrophages. The importance of IFNG in immunology derives from its ability to inhibit viral replication directly and exert immunostimulatory and immunomodulatory effects. IFNG is produced by natural killer and natural killer T cells in innate immune responses and by CD4 Th1 and CD8 cytotoxic T lymphocyte effector T cells after development of antigen-specific immunity [14]. IFNG induces cellular responses via its interaction with a heterodimeric receptor consisting of IFNG receptor 1 (IFNGR1) and IFNGR2 which activates the JAK-STAT pathway. IFNG also binds to heparan sulfate at the cell surface which inhibits its biological activity [14].

### Characteristics of interferon Tau

IFNT was discovered by culturing sheep conceptuses in the presence of radiolabeled amino acids and detecting radiolabeled *de novo* synthesized proteins that included an abundant low molecular weight protein first named protein X and then ovine trophoblast protein 1 [15-18] in my laboratory and trophoblastin by Martal et al. [19]. When the gene for oTP1/trophoblastin was cloned and sequenced it was found to be a type 1 interferon designated IFNT [20,21]. The antiviral, antiproliferative and immunosuppressive activities, and insight into its structural motif have been reported [22-25]. My laboratory used a synthetic gene for IFNT to produce recombinant IFNT with immunosuppressive, antiviral, antiproliferative and antiluteolytic properties identical to those for native IFNT [26,27]. IFNT has a molecular weight of 19 to 24 kDa depending on glycosylation and an isoelectric point between 5.3 and 5.8. It has 172 amino acids with disulfide bridges between cysteine residues at 1 and 99, as well as 29 and 139 [28]. Ovine IFNT is not glycosylated, whereas bovine IFNT is N- glycosylated and caprine IFNT is a mixture of nonglycosylated and N-glycosylated forms with the glycosylation site being at ASN 78. The amino terminal amino acid is proline. IFNT is very stable to pH as low as 2 to 3 [28].

### Antiluteolytic effects of interferon Tau

**Figure 1:**
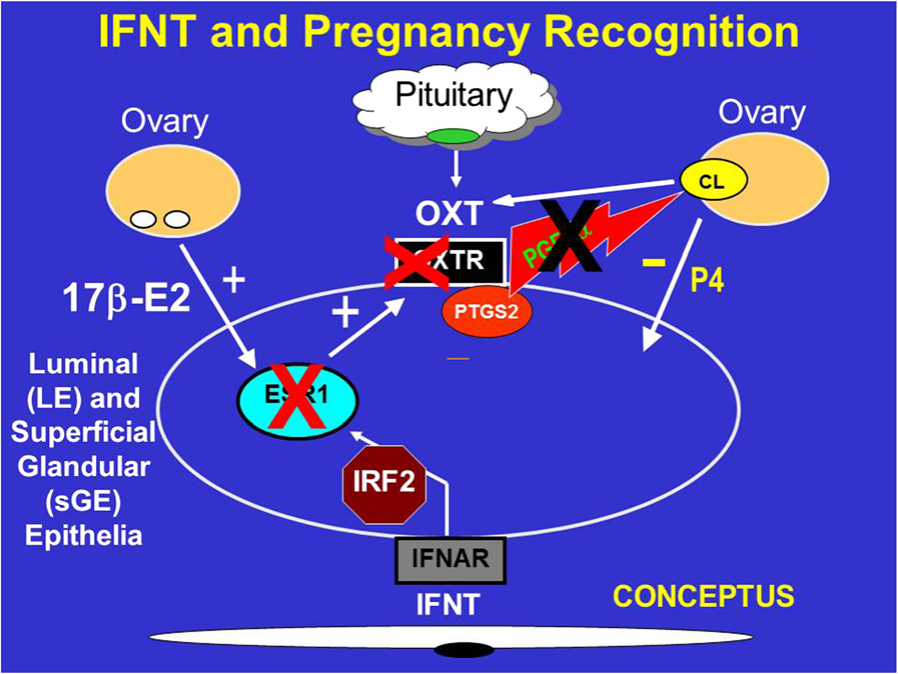
**Interferon tau (IFNT) is the pregnancy recognition hormone in sheep and other ruminants that acts to silence expression of estrogen receptor alpha (ESR1) and, in turn, oxytocin receptor (OXTR) to prevent development of the luteolytic mechanism that required oxytocin (OXT) from the corpus luteum (CL) and posterior pituitary to induce luteolytic pulses of prostaglandin F_2α_ (PGF).** Thus, IFNT blocks the ability of the uterus to develop the luteolytic mechanism, but does not inhibit prostaglandin synthase 2 (PTGS2) or the basal production of PGF during pregnancy.

The model for studies of the antiluteolytic effect of IFNT in my laboratory was based on McCracken’s model of the “progesterone block” for regulation of the estrous cycle in ewes [29]. The hypothesis states that P4 blocks expression of estrogen receptor alpha (ESR1) and oxytocin receptor (OXTR) for about 10 days after which time P4 down-regulates expression of progesterone receptors (PGR) in uterine epithelia which allows rapid increases in expression of *ESR1* and *OXTR* genes (Figure 1). Then, pulsatile release of oxytocin (OXT) from the posterior pituitary gland and CL induce pulsatile secretion of prostaglandin F2α (PGF) from uterine epithelia on Days 15 and 16 which induces functional and structural regression of the CL followed by estrus and another opportunity for the ewe to mate and become pregnant. Our understanding of pregnancy recognition in ruminants is from studies see [30-32] indicating that: 1) IFNT silences transcription of the *ESR1* gene and, therefore, estradiol-induced expression of *OXTR* in uterine luminal and superficial glandular epithelia (LE/sGE) to abrogate development of the endometrial luteolytic mechanism involving OXT-induced luteolytic pulses of PGF; 2) basal production of PGF and PGE2 is higher in pregnant than cyclic ewes due to continued expression of prostaglandin synthase 2 (*PTGS2*) in uterine LE/sGE; 3) IFNT silencing of *ESR1* expression prevents estradiol from inducing PGR in endometrial epithelia; and 4) loss of PGR by uterine epithelia is required for expression of P4-induced and IFNT-stimulated genes that support development of the conceptus. Caprine IFNT secreted between Days 16 and 21 of gestation also abrogates the luteolytic mechanism to prevent pulsatile release of luteolytic PGF and extend lifespan of the CL in goats [33]. Bovine IFNT, secreted between Days 12 and 38 of pregnancy, also prevents secretion of luteolytic pulses of PGF by uterine epithelia and blocks effects of exogenous E2 and oxytocin to stimulate uterine release of PGF. Expression of *ESR1* and *OXTR* mRNAs is either silenced or the receptors are not responsive to estradiol and OXT in endometria of both pregnant cows and cyclic cows treated with intrauterine injections of either ovine or bovine recombinant or native IFNT see [34,35].

**Figure 2:**
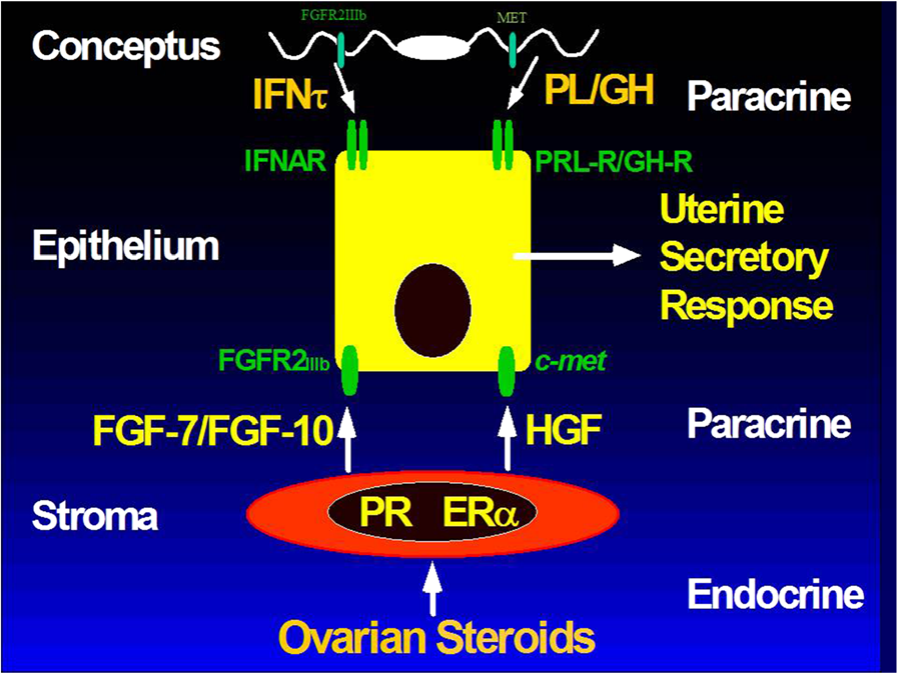
**Silencing expression of progesterone receptor (PGR) in uterine epithelia is a prerquisite for implantation in mammals.** Therefore, progesterone (P4) acts via PGR-positive uterine stromal cells to increase expression of progestamedins, e.g. fibroblast growth factor-7 (FGF7) and FGF10, as well as hepatocyte growth factor (HGF) in sheep uteri. The progestamedins, as well as interferon tau (IFNT) exert paracrine effects on uterine epithelia and conceptus trophectoderm that express receptors for FGF7 and FGF10 (FGFR2_IIIb_) and HGF (MET) to stimulate cell signaling pathways including phosphatidyl inositol kinase 3 kinase (PI3K) and mitogen activated protein kinase (MAPK) to stimulate gene expression and secretory responses by trophectoderm and uterine luminal (LE) and superficial glandular (sGE) epithelia that do not express signal transducers and activators of transcription (STAT1/STAT2). Thus, IFNT activates undefined alternate cell signaling pathways that may include PI3K and MAPK to influence gene expression by uterine LE and sGE.

IFNT silences expression of ESR1 to ensure that estradiol does not increase expression of ESR1 in uterine epithelia during pregnancy. Thus, uterine LE/sGE do not express ESR1, PGR, IRF9 or STAT1 because IFNT induces expression of IRF2, a potent suppressor of transcription, in uterine LE/sGE that is in direct contact with conceptus trophectoderm [31]. Therefore, uterine LE/sGE in direct contact with the conceptus express unique non-classical interferon stimulated genes such as those for transport of nutrients into the uterine lumen to support growth and development of the conceptus. The uterine LE/sGE are affected by P4; however, the action of P4 is mediated by PGR-positive uterine stromal cells that secrete one or more progestamedins, particularly FGF10 in ewes, and effects of IFNT on uterine LE/sGE are mediated via a JAK/STAT-independent cell signaling pathway [3,30,31]. Therefore, IFNT abrogates the uterine luteolytic mechanism to prevent pulsatile release of luteolytic PGF while also increasing expression of many genes critical for uterine receptivity to implantation and conceptus development (Figure 2). These genes include wingless-type MMTV integration site family member 7A (*WNT7A*) induced by IFNT, as well as *LGALS15* (galectin 15), *CTSL* (cathepsin L), *CST3* (cystatin C), *SLC2A1* (solute carrier family 2 (facilitated glucose transporter), member 1), *SLC7A2* (cationic amino acid transporter), HIF2A (hypoxia-inducible factor 2A) and gastrin releasing peptide (GRP) that are induced by P4 and further stimulated by IFNT and/or prostaglandins [30].

### Prostaglandins and IFNT affect uterine gene expression and conceptus development

Dorniak et al. [36] reported that prostaglandins (PG) secreted by epithelial and stromal cells of the uterus effect expression of genes critical to elongation and implantation of the ovine conceptus. Although IFNT inhibits expression of ESR1 and OXTR in uterine LE/sGEof pregnant ewes, IFNT does not inhibit expression of prostaglandin synthase 2 (PTGS2), the rate-limiting enzyme in synthesis of PGs. IFNT stimulates PGE2 production by cells of the bovine uterus and other Type I IFNs stimulate phospholipase A2 (PLA2) and synthesis of PGE2 and PGF in various cell types. Intra-uterine infusions of meloxicam, a specific inhibitor of PTGS2, prevents elongation of ovine conceptuses. The elongating conceptuses of ewes and cows synthesize and secrete more PGs than the uterus; therefore, the abundance of PGs is greater in the uterine lumen of pregnant as compared to cyclic ewes and cows. Sheep conceptuses secrete mainly PGF, 6-keto-PGF1α (i.e., a stable metabolite of PGI2), and PGE2 during the peri-implantation period of pregnancy and PG receptors are present in all cell types of the uterus and conceptus during pregnancy. Conceptus-derived PGs have autocrine, paracrine and possibly intracrine effects on cells of the uterus and conceptus. For example, the expression of PTGS2 by Day 7 bovine blastocysts predicts successful development of that blastocyst to term and delivery of a live calf. The infusion of PGE2, PGF, PGI2 or IFNT into the uterine lumen of cyclic ewes increases expression of GRP, insulin-like growth factor binding protein 1 (IGFBP1) and LGALS15, but only IFNT increases expression of cystatin 6 (CST6). Differential effects of PGs were also observed for CTSL and its inhibitor CST3. For glucose transporters, IFNT and all PGs increased SLC2A1, but only PGs increased SLC2A5 expression, whereas expression of *SLC2A2* and *SLC5A1* mRNAs were increased by IFNT, PGE2, and PGF. Infusions of all PGs and IFNT increased the amino acid transporter SLC1A5, but only IFNT increased SLC7A2. In the uterine lumen, only IFNT increased glucose concentrations, and only PGE2 and PGF increased the abundance of total amino acids. Thus, PGs and IFNT coordinately regulate endometrial functions important for growth and development of the conceptus during the peri-implantation period of pregnancy.

### Cortisol regulates endometrial function

The expression of 11-beta-hydroxysteroid dehydrogenase, type I (HSD11B1) is induced by P4 and stimulated by IFNT in ovine uterine LE/sGE and it is one of two isoforms that regulate intracellular levels of bioactive glucocorticoids. The ovine uterine endometrium and conceptus generate active cortisol from inactive cortisone and cortisol regulates expression of genes via the glucocorticoid receptor (GR). The few GR target genes identified in the uterus or placenta include those involved in lipid metabolism and triglyceride homeostasis. In addition to progesterone induction and IFNT stimulation of HSD11B1 expression in the ovine endometrium, PGs regulate activity of HSD11B1 in the bovine endometrium, and PGF stimulates HSD11B1 activity in human fetal membranes [36-38]. Elongating sheep conceptuses generate cortisol from cortisone via HSD11B1. GR are present in all cells of ovine uterus during the estrous cycle and pregnancy and in conceptus trophectoderm; therefore, cortisol may have paracrine and autocrine effects on the endometrium and conceptus trophectoderm. Intrauterine infusions of cortisol into cyclic ewes from Days 10 to 14 increased expression of several elongation- and implantation-related genes in ovine uterine epithelia. In humans, cortisol at the conceptus-maternal interface is proposed to stimulate secretion of chorionic gonadotropin by trophoblast, promote trophoblast growth and invasion, and stimulate placental transport of glucose, lactate, and AA. Interestingly, administration of glucocorticoids increased pregnancy rates in women undergoing assisted reproductive technologies and pregnancy outcomes in women with a history of recurrent miscarriage [39,40].

### Interferon Tau drives a servomechanism for uterine functions

The establishment and maintenance of pregnancy requires integration of endocrine and paracrine signals from the ovary, conceptus, and uterus [41]. In ewes, implantation and placentation occur as a protracted process from Days 15–16 to Days 70 to 80 of pregnancy [42,43]. During this period, the uterus and placenta grow and remodel for support of rapid conceptus development and growth during the last one-half of pregnancy [44]. In addition to development of placentomes in the caruncular areas of the endometrium and changes in uterine vascularity, the uterine glands in the intercaruncular endometrium increase in length (4-fold) and width (10-fold) and degree of secondary and tertiary branching during pregnancy [42]. Hyperplasia of uterine GE occurs between Days 15 and 50 to 60 of gestation and then uterine glands undergo hypertrophy to increase surface area for maximal production of histotroph after Day 60 [45].

The ovine uterus is exposed sequentially to estrogen, progesterone, IFNT, placental lactogen (CSH1), and placental growth hormone (GH1) during pregnancy as these hormones initiate and maintain endometrial gland morphogenesis and differentiated secretory functions of uterine GE [46]. Ovine CSH1 is produced by binucleate cells of conceptus trophectoderm from Days 15 or 16 of pregnancy which is coordinate with onset of expression of genes for uterine milk proteins (UTMP) and secreted phosphoprotein 1 (SPP1, also known as osteoponin) by uterine GE [45,47]. UTMP are members of the serpin family of serine protease inhibitors [48] and SPP1 is an extra-cellular matrix protein [49]. UTMP and SPP1 are excellent markers for differentiation and overall secretory capacity of uterine GE during pregnancy in ewes [46]. CSH1 is detectable in maternal serum by Day 50 and peak concentrations are between Days 120 to 130 of gestation [50]. A homodimer of the prolactin receptor (PRLR) and a heterodimer of PRLR and growth hormone receptor (GHR) transduce CSH1 cell signaling [51]. In the ovine uterus, CSH1 binding sites for PRLR are specific to GE [52]. Temporal changes in circulating levels of CSH1 are correlated with endometrial gland hyperplasia and hypertrophy and increased production of UTMP and SPP1 during pregnancy [45,49]. Placental GH1 is produced between Days 35 and 70 of gestation [53] when onset of hypertrophy of uterine GE occurs along with maximal increases in the abundance of UTMP and SPP1 proteins from uterine GE. Thus, two members of the lactogenic and somatogenic hormone family stimulate endometrial gland morphogenesis and differentiated function during pregnancy to facilitate conceptus growth and development in ewes.

The sequential exposure of the ovine uterus to estrogen, progesterone, IFNT, CSH1 and placental GH1 during pregnancy constitutes a “servomechanism” that activates and maintains remodeling, secretory function and growth of the uterus [46]. Chronic treatment of ovariectomized ewes with progesterone induces expression of UTMP and CSH1 by uterine GE and insures that PGR are not in uterine epithelia beyond Day 13 post-estrus [41]. Down-regulation of PGR in uterine GE is required for progesterone to induce expression of UTMP and SPP1, but a combination of progesterone and estrogen increases expression of ESR1 and PGR in uterine GE which inhibits expression of both *SPP1* and *UTMP*. Thus, progesterone must down-regulate expression of PGR in uterine GE in order for CSH1 and GH1 to stimulate expression of *UTMP* and *SPP1*[46].

The intrauterine infusion of CSH1 or GH1 increases expression of *UTMP* and *SPP1* by uterine GE of progesterone-treated ewes. However, the ewes must first receive intrauterine infusions of IFNT between Days 11 and 21, and then either CSH1 or GH1 from Days 16 to 29 after onset of estrus [46]. The increase in expression of *UTMP* by uterine GE is due in part to effects of CSH1 and GH1 to increase branching and surface area of uterine glands. Intrauterine infusion of CSH1 and GH1 into ewes treated with progesterone and IFNT increased hypertrophy of uterine glands, but this response did not occur if ewes were not treated with IFNT prior to receiving intra-uterine infusions of CSH1 or GH1. The ability of prolactin, CSH1 and GH1 to elicit similar effects on uterine glands is consistent with the fact that these hormones are members of a unique hormone family that shares genetic, structural, binding, receptor signal transduction and function on glandular tissues including the uterus and mammary gland [51]. These studies revealed that developmentally programmed events mediated by specific paracrine-acting hormones at the conceptus-uterine interface stimulate remodeling and differentiated function of uterine GE for production of histotroph essential for fetal-placental growth during gestation. Importantly, actions of IFNT, through an unknown mechanism, are required for actions of CSH1 and GH1 on uterine gland development and function.

### Pregnancy recognition signaling in pigs

**Figure 3:**
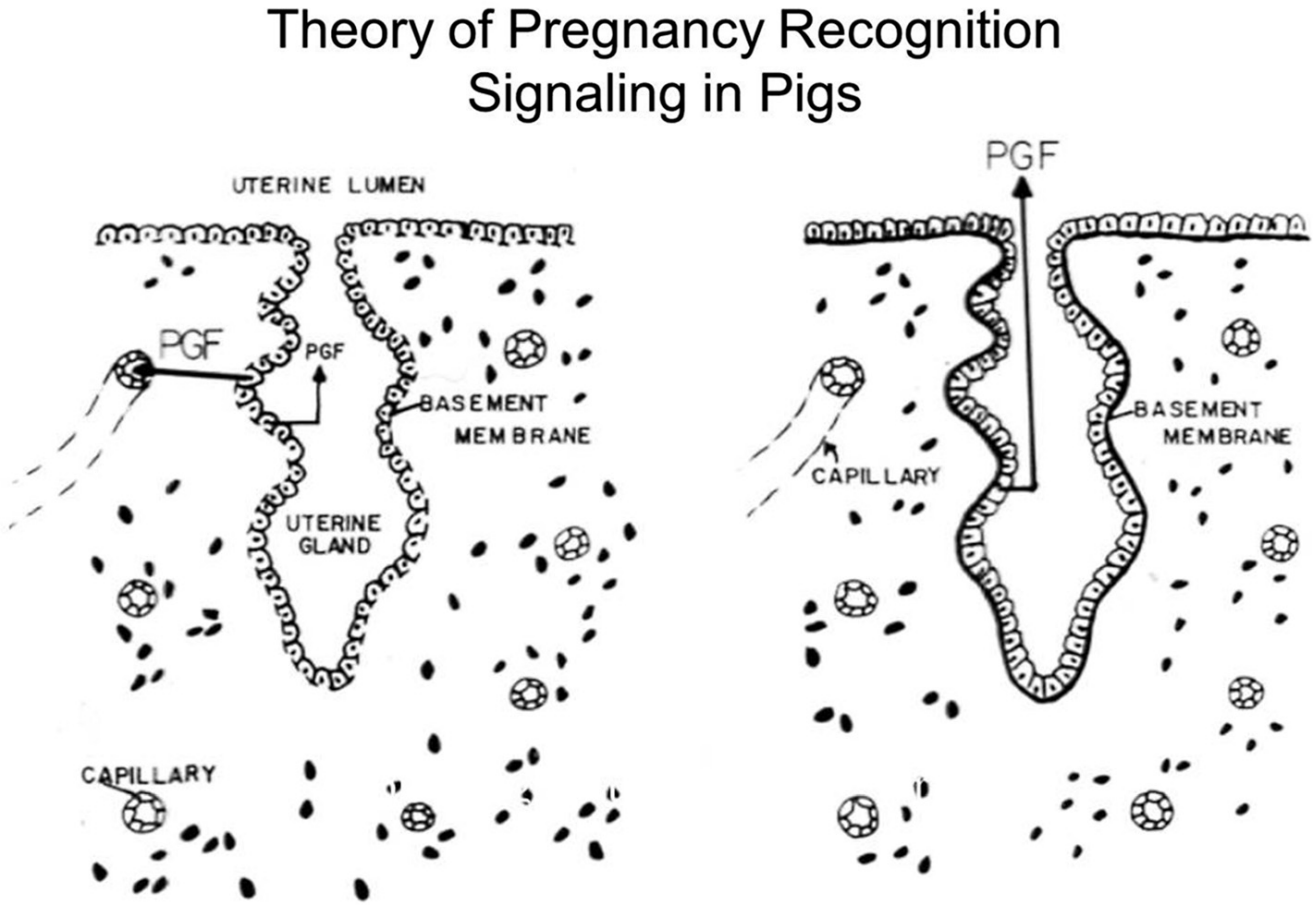
**The theory of pregnancy recognition in the pig is that secretion of prostaglandin F_2α_ (PGF) is endocrine, that is, toward the uterine vascular drainage to induce luteolysis in cyclic pigs.** However, PGF is secreted in an exocrine direction, that is, toward the uterine lumen in pregnant pigs where it is metabolized and unavailable to exert luteolytic effects.

The blastocysts of pigs undergo a morphological transition from large spheres of 10 to 15 mm diameter and then tubular (15 mm by 50 mm) and filamentous (l mm by 100–200 mm) forms between Days 10 and 12 of pregnancy and achieve a length of 800 to 1000 mm between Days 12 and 15 of pregnancy see [31]. Rapid elongation of conceptus trophectoderm allows maximum surface area of contact between trophectoderm and uterine LE/sGE. During this period of rapid elongation, the trophectoderm secretes estrogens (catecholestrogens, estrone and estradiol) [54], and IFNG and IFND [4,5]. Estrogen is the pregnancy recognition signal from conceptus trophectoderm in pigs and it must be secreted between Days 11 and 15 of pregnancy. Estrogen does not inhibit secretion of PGF by uterine endometrium, rather it activates a mechanism whereby secretion of PGF is into the uterine lumen (exocrine secretion) rather than into the uterine vasculature (endocrine secretion) as occurs in nonpregnant gilts and sows (Figure 3). Thus, in pregnant pigs, PGF is sequestered within the uterus and metabolized to prevent it from exerting luteolytic effects on the CL. The conceptus estrogens also modulate expression of genes responsible for endometrial remodeling for implantation between Days 13 and 25 of gestation [55]. Both SPP1 and FGF7 are induced by estrogen in uterine LE to affect trophectoderm and LE adhesion, signal transduction and cell migration during the peri-implantation period [56-58]. The trophectoderm also secretes interleukin 1 beta (IL1B) during this period and estrogen appears to modulate uterine responses to IL1B [59].

Pig conceptus trophectoderm secretes both IFNG and IFND during the peri-implantation period of pregnancy [4,5]. *IFNG* mRNA is abundant in trophectoderm between Days 13 and 20 of pregnancy, whereas *IFND* mRNA is detectable in Day 14 conceptuses only by RT-PCR analysis [54]. IFNG and IFND proteins co-localize to peri-nuclear membranes typically occupied by the endoplasmic reticulum and golgi apparatus, as well as cytoplasmic vesicles within clusters of trophectoderm cells along the uterine LE. This expression is characterized by *de novo* appearance of zona occludens one (ZO1), a marker of epithelial tight junctions on their basal aspect which suggests changes in endometrial polarity [5]. There is no evidence that either IFNG or IFND have antiluteolytic effects to prevent regression of CL or alter concentrations of progesterone in plasma. However, they do stimulate secretion of PGE2 by uterine cells which may enhance structural integrity of CL and their secretion of P4 [60].

A number of genes are expressed by uterine epithelial and stromal cells in pigs in response to intra-muscular injections of estradiol and/or intra-uterine injections of pig conceptus secretory proteins that include IFNG and IFND [61-63]. Implantation in pigs is non-invasive and pigs have a true epitheliochorial placenta. Genes induced in uterine LE by estrogen include *SPP1*, *FGF7*, aldo-keto reducing family 1 member B1 (*AKR1B1*), cluster of differentiation 24 (*CD24*), neuromedin beta (*NMB*), *STAT1* and *IRF2*. Expression of IRF2 is induced in uterine LE/sGE by estrogen, the pregnancy recognition signal in pigs whereas IFNT induces IRF2 in uterine LE/sGEin ewes. In both pigs and ewes the expression of IRF2 in uterine LE and sGE prevents IFNT in ewes and IFNG and IFND in pigs from inducing expression of ISG in uterine LE/sGE. The genes expressed by uterine LE of pigs are for stimulation of proliferation, migration and attachment of trophectoderm to uterine LE. Also, IFND and/or IFNG may affect blastocyst attachment to uterine LE in pigs by inducing labilization and remodeling of uterine LE to affect polarity and stimulate production of PGE2.

Since IRF2 is expressed in uterine LE of pigs, these cells do not express classical ISG, rather expression of classical ISG is limited to uterine GE and stromal cells [66]. The classical ISG induced by IFNG and/or IFND in uterine GE and stromal cells, as well as endothelial cells include STAT1, STAT2, IRF1, MX1, swine leukocyte antigens (SLA) 1–3 and 6–8, and beta 2 microglobulin. The pregnancy-specific roles of these uterine ISGs may be to: 1) affect decidual/stromal remodeling to protect the fetal semi-allograft from immune rejection; 2) limit conceptus invasion into the endometrium; and/or 3) stimulate development of uterine vasculature. Because IFNG can initiate development of the endometrial vasculature, it is hypothesized to facilitate establishment of hematotrophic support of developing conceptuses.

Secretion of both IFND and IFNG by conceptus trophectoderm is unique to pig conceptuses, but little is known of their interactions. Type I IFND and Type II IFNG may each induce expression of non-overlapping sets of genes; however, they may act synergistically to induce physiological responses. Cooperative induction and maintenance of expression of ISGs such as STAT1 for reinforcement of their effects on distinct cell-surface ligands while maintaining their individual specificities for inducing ISGs may occur. Although IFNG may enhance uterine receptivity to implantation in pigs, highly localized and abundant expression of IFNG, TNFA, IL1B and IL1R in the endometrium is reported to interfere with conceptus development between Days 15 and 23 of pregnancy [64].

#### Progestamedins, estramedins, corticoids and prostaglandins

Uterine receptivity to implantation is dependent on progesterone which is permissive to actions of IFNs, chorionic gonadotrophin and lactogenic hormones such as prolactin and placental lactogen [2,30-32]. The paradox is that cessation of expression of PGR and ESR1 by uterine epithelia is a prerequisite for uterine receptivity to implantation, expression of genes for secretory proteins by uterine epithelia, and selective transport of molecules into the uterine lumen that support conceptus development. Down-regulation of PGR is associated with loss of expression of proteins on uterine LE such as MUC1 which would interfere with implantation. Further, silencing expression of *PGR* in uterine epithelia allows progesterone to act on PGR-positive uterine stromal cells to induce expression of progestamedins, i.e., FGF7 and −10, and hepatocyte growth factor (HGF), that exert more specific paracrine regulation of differentiated functions of uterine epithelia and conceptus trophectoderm that express receptors for FGF7 (FGFR2IIIb) in pigs see [30]. Many ISGs are P4-induced and IFN-stimulated; however, a fundamental unanswered question is whether actions of progestamedins and IFNs on uterine epithelia or other uterine cell types involve non-classical cell signaling pathways, independent of PGR and STAT1, such MAPK and PI3K/AKT to affect gene expression and uterine receptivity to implantation [1,30]. Interestingly, type I IFNs bind the same receptor, but activate unique signaling pathways that are cell-specific to differentially affect gene expression in uterine LE/sGE versus GE and stromal cells [55,64,65].

### Estramedins in pigs

Pig conceptuses secrete estrogens between Days 10 and 15 for pregnancy recognition, but also to increase expression of growth factors including insulin-like growth factor 1 (IGFI) and FGF7 which, in turn, act on conceptus trophectoderm to stimulate proliferation and/or gene expression [32]. *IGFI* is expressed by uterine glands of cyclic and pregnant pigs and IGF1 receptors (*IGF1R*) are expressed by cells of the endometrium and conceptuses suggesting paracrine and autocrine actions of IGFI. *FGF7*, an established paracrine mediator of hormone-regulated epithelial growth and differentiation, is expressed uniquely by uterine LE in pigs between Days 12 and 20 of the estrous cycle and pregnancy. FGF7 binds to and activates FGFR_2IIIb_ expressed by uterine epithelia and conceptus trophectoderm. Estradiol increases *FGF7* expression following effects of progesterone to down-regulate expression of PGR in uterine LE. FGF7 then increases cell proliferation, phosphorylated FGFR2IIIb, the MAPK cascade and expression of urokinase-type plasminogen activator, a marker for trophectoderm cell differentiation [56,59]. From about Day 20 of pregnancy, FGF7 expression shifts from uterine LE to uterine GE in pigs and likely continues to affect uterine epithelia and conceptus development [57,58]. In addition to the increase in secretion of estrogens between Days 11 and 15 of pregnancy for maternal recognition of pregnancy, increases in estrogens from the placenta between Days 20 and 30 increase expression of endometrial receptors for prolactin that may allow prolactin to stimulate secretions from uterine GE, placentation and uterine blood flow for increased transport of nutrients [66].

### Corticoids

There are positive actions of glucocorticoids in early pregnancy. For example, in primates, glucocorticoids stimulate secretion of chorionic gonadotrophin, suppressuterine natural killer cells, and promote trophoblast growth and invasion, as well as exert negative effects that might compromise pregnancy that include inhibiting cytokine-prostaglandin signaling, restriction of trophoblast invasion, induction of apoptosis, and inhibition of conceptus development [67]. With respect to implantation of blastocysts, a dialogue initiated by cell surface signalling molecules on conceptus trophectoderm and uterine LE includes integrins and fibronectin that glucocorticoids suppress to enhance implantation. The effects of glucocorticoids on fibronectin expression are tissue-specific with dexamethasone suppressing fibronectin in term human cytotrophoblasts and amnion, but acting in synergy with transforming growth factor beta to increase expression of fibronectin in matched samples of chorion and placental mesenchymal cells. Also occurring during the peri-implantation period of pregnancy are events mediated by pro-inflammatory cytokines such as IL1B, TNFA and prostaglandins that are modulated by anti-inflammatory effects of glucocorticoids which likely modulate cytokine-prostaglandin signaling required for implantation. Both IL1B and TNFA increase expression and activity 11BHSD1 while suppressing expression of 11BHSD2 in term human chorionic trophoblasts. This has the net effect of increasing the conversion of corticosterone to cortisol and creating a negative feedback loop at the uterine-conceptus interface between glucocorticoids and inflammatory cytokines.

In most tissues, one aspect of the anti-inflammatory effect of glucocorticoids is to inhibit the synthesis of prostaglandins and thromboxanes by decreasing the expression and/or actitivity of phospholipase A2 (PLA2) and, therefore, liberation of arachidonic acid as substrate for PTGS1 and PTGS2 [68]. However, in the placenta, glucocorticoids increase PLA2, PTGS2 and prostaglandin synthases [69] and decrease expression of 15-alpha hydroxyprostaglandin dehydrogenase (HPGD) that converts prostaglandins to their inactive forms [70]. Within the placenta, prostaglandins increase expression and activity of 11BHSD1 [37] to increase cortisol production and decrease activity of 11BHSD2 that converts cortisol to inactive cortisone [71]. Glucocorticoids can stimulate growth of trophoblast and expression of pro-matrix metalloproteinase (proMMP-2) [72], but other reports indicate that they inhibit expression of MMP9 and migration (invasiveness) of cytotrophoblast cells [73]. Further, glucocorticoids affect degradation of extracellular matrix during trophoblast invasion with urokinase-type plasminogen activator (uPA) that leads to plasmin-associated degradation of extracellular matrix and tissue-type enzyme (tPA) plasmin-dependent breakdown of fibrin for establishment of an efficient vascular exchange in the placenta [74]. The activities of both uPA and tPA are inhibited by plasminogen activator inhibitor (PAI1) secreted by trophoblast and decidual cells [75] and both cortisol and dexamethasone increase expression of PAI1 [76] which may result in poor placental exchange of nutrients and gases and lead to pre-eclampsia and intra-uterine growth retardation [77].

In sheep, establishment of pregnancy requires elongation of the conceptus and production of IFNT for pregnancy recognition signaling as discussed previously. Expression of HSD11B1 may be stimulated by P4, prostaglandins and/or cortisol and *HSD11B1* mRNA is more abundant in uterine LE/sGE between Days 12 and 16 of pregnancy than the estrous cycle and expression of both HSD11B1 and PTGS2 by uterine LE/sGE is coordinate with conceptus elongation in ewes [78]. Physiological levels cortisol are also potent stimulators of expression of both arginase and ornithine decarboxylase in cells which increases synthesis of polyamines essential for cell proliferation and differentiation of cells of the conceptus [79]. Although HSD11B1 is abundant in the uterine epithelia, it is barely detectable in the conceptus, whereas HSD11B2 is barely detectable in uterine epithelia, butabundant in the conceptus. Expression of HSD11B1 is induced by P4 and further stimulated by IFNT in uterine LE/sGE. The corticoid receptor, NR3C1, is present in all ovine uterine cell types. Therefore, HSD11B1 expression in uterine LE/sGE is regulated by P4, IFNT and prostaglandins generate cortisol that act via NR3C1 to regulate ovine endometrial functions, such as production of prostaglandins, during pregnancy. Prostaglandins represent another activator of gene expression via their respective receptors, such as PGE receptors (PTGER1-PTGER3) to activate MAPK cell signaling pathways. In bovine uteri, IFNT stimulates expression of PTGS2 and PGE synthase to increase the relative abundance of PGE, but also increases expression of prostaglandin E receptor 2, EP2 subtype in endometrial epithelia [80] and PGE may stimulate gene expression by activation of p38 MAPK [81]. Therefore, in uterine epithelia, there is the potential for IFNT, progestamedins and prostaglandins to act additively or synergistically to stimulate expression of genes by uterine epithelia that support growth and development of the conceptus.

## Summary

The focus of this review is pregnancy recognition signaling molecules in ruminants by IFNT and in pigs by estrogens. IFNT abrogates development of the luteolytic mechanism by silencing expression of ESR1 and OXTR to prevent pulsatile release of luteolytic PGF by uterine epithelia. Estrogens from pig conceptuses, on the other hand, induce mechanisms for exocrine secretion of PGF into the uterine lumen where is metabolized and, therefore, unavailable to cause luteolysis. Both IFNT and estrogens, in concert with effects of progesterone, exert effects particularly on uterine LE and sGE the increase expression of genes that include growth factors and nutrient transporters critical to growth and development of the conceptus. The PGs and corticoids within the uterine lumen also play important roles in regulation of gene expression favorable to a uterine environment supportive of conceptus development. The complex interactions between hormones from the ovaries, conceptus trophectoderm/placenta and maternal pituitary are discussed with respect to effects on growth and development of uterine glands that secretion of nutrients critical to conceptus development. Collectively, the outcome of actions of the many hormones, growth factors, cytokines, lymphokines, extra-cellular matrix and nutrients is highly conducive to a successful outcome of pregnancy that includes establishment of mechanisms whereby the conceptus semi-allograft is protected from the maternal immune system.

## Competing interests

The author has nothing to declare regarding conflicts of interest or competing financial interests.
